# Evaluation of evidence-based medicine adoption among Nigerian surgeons: competence, knowledge, attitudes, practices, and barriers

**DOI:** 10.3325/cmj.2024.65.3

**Published:** 2024-02

**Authors:** Kehinde Oluwadiya, Anthony Olasinde, Ibironke Oluwadiya, Johnson Ogunlusi, Eyitayo Alabi

**Affiliations:** 1 Department of Surgery, Ekiti State University, Ado-Ekiti, Ekiti State, Nigeria; 2 Department of Surgery, Kampala International University, Ishaka-Bushenyi, Uganda; 3 Department of Demography and Social Statistics, Obafemi Awolowo University, Ile-Ife, Osun State, Nigeria; 4 Department of Surgery, Ekiti State College of Medicine, Ado-Ekiti, Ekiti State, Nigeria; 5 Department of Surgery, College of Medicine, University of Lagos, Lagos, Nigeria

## Abstract

**Aim:**

To determine the competence, knowledge, attitude, and barriers to evidence-based medicine (EBM) among surgeons in southwest Nigeria.

**Methods:**

Between April 1 and June 30, 2019, a composite questionnaire consisting of the McAllister knowledge and attitude questionnaire, the Zwolsman barrier questionnaire, and 5 questions from the Berlin Questionnaire (BQ) on competence in EBM was administered to 185 surgeons and trainees in five hospitals in southwest Nigeria.

**Results:**

The study involved 169 respondents (57 surgeons and 112 trainees). A total of 122 (72.1%) participants reported to always/often use EBM in their practice and 47 (27.9%) to rarely/never use EBM. The majority of both groups still relied on traditional sources of information in their clinical practice. Even though self-identified EBM-users (28% points) scored significantly higher on the BQ than non-users (23.8% points), there was no difference in their performance on the McAllister and Zwolsman questionnaires. Paradoxically, those with prior training in EBM were not more likely to use EBM than those without training, and there were no significant differences in their BQ scores.

**Conclusions:**

Surgeons in Nigeria have a high level of awareness and use of EBM, as indicated by the 72% awareness rate found in our study, but their knowledge and confidence in its application are low. Our findings indicate that the quality of EBM training in the region needs to be reevaluated.

Evidence-based medicine (EBM) is an approach to health care that emphasizes the use of evidence from high-quality research to inform clinical decision-making. It involves asking clinically relevant questions, finding the evidence, appraising the evidence, and applying the evidence in practice. By following these steps, health care professionals can incorporate the latest research into their practice, which leads to improved patient outcomes and increased efficiency in the health care system (1).

EBM is relatively new, with its first recorded use in medical literature dating back to 1992. Since then, it has been widely adopted in most parts of the world (2,3). In many parts of the world, it has been incorporated into both undergraduate (4) and postgraduate medical education (5), and many countries have established official guidelines and online resources to guide EBM practice, such as NICE in the UK (6), AHRQ in the USA (7), and AAZ in Croatia (8).

Despite its widespread acceptance in many parts of the world, the adoption of EBM in developing countries - and by certain specialties, such as surgery - has been slower (9-14). For instance, the first publication from Nigeria on EBM appeared only in 2008 (14). In these regions, limited resources and access to current research pose significant challenges to implementing EBM. The global movement toward EBM thus highlights disparities in health care practices between developed and developing nations.

In the context of Nigeria, the adoption of EBM faces unique challenges (10,11,13). For example, there are no national guidelines on EBM in Nigeria comparable to those in the UK, Croatia, or USA. Moreover, EBM is not integrated into the most current guidelines for medical education in Nigeria set by the National University Commission and the Medical and Dental Council of Nigeria (15). As a result, the adoption of EBM in Nigeria is currently driven by individual interests and specialties.

Among the specialties, the surgical specialty has been slower than most specialties in embracing EBM (16). One reason for this is that it is difficult to evaluate the “art of surgery” in randomized controlled trials, which are considered the gold standard of evidence in EBM (9). Additionally, surgeons and indeed other physicians often cite practical difficulties such as the time and effort required to search for and critically appraise the literature. Furthermore, there are concerns raised in the literature about EBM potentially interfering with patient individuality and surgeon autonomy (9,17).

Thus, the Nigerian surgeon faces two distinct disadvantages regarding the adoption of EBM. The first is the lack of national guidelines, and the second is the surgical profession's reluctance to adopt EBM. These factors underscore the importance of examining the understanding, acceptability, and barriers to the adoption of EBM among surgeons in Nigeria. Consequently, the aim of this study is to explore the competence, knowledge, attitudes, practices, and perceived barriers to EBM among surgeons and surgical trainees in Nigeria. We hypothesized that surgeons and surgical trainees in Nigeria would exhibit varied levels of competence, knowledge, attitudes, and practices concerning EBM, which are significantly influenced by the absence of national guidelines and support structure for the adoption of EBM in the country.

## Methods

### Study design

In this cross-sectional study, the data collection involved the use of a self-administered questionnaire (https://osf.io/674uz/), completed between April 1 and June 30, 2019. A convenience sampling was performed in such a way that each respondent was approached by one of the researchers or a research assistant and given the option to complete the questionnaire either online or in a paper format. For those who chose the paper format, research assistants distributed the questionnaires for respondents to fill out and scheduled a time to collect the completed forms. Participants opting to complete the questionnaire online were provided with a link to the questionnaire hosted on Google Forms, and the researcher or the research assistant followed up at least once to remind them.

### Participants and setting

The study participants were surgeons and surgical trainees from five tertiary hospitals in four states in Southwestern Nigeria: Ekiti State University Teaching Hospital Ado-Ekiti and the Federal Teaching Hospital, Ido-Ekiti in Ekiti State; Obafemi Awolowo Teaching Hospitals Complex, Ile-Ife in Osun State; LAUTECH Teaching Hospital Ogbomosho in Oyo State; and University of Ilorin Teaching Hospital, Ilorin, in Kwara State. There were 131 (42.2%) consultants and 180 (57.8%) resident doctors in the five hospitals (a total of 311).

In Nigeria, surgical residency training is split into a three-year junior and a three-year senior program. While the training is hospital-based, it is supervised by the two accredited postgraduate medical colleges in Nigeria, which offer periodic workshops and training modules in various aspects of surgical theory and practice. However, none of these training modules focus on EBM. EBM is not mentioned in the “Benchmark for Minimum Academic Standards for Undergraduate Programs in Medical Education” of the Nigerian Universities Commission or in the “Guideline on Minimum Standards of Medical and Dental Education in Nigeria” of the Medical and Dental Council of Nigeria, which are the accrediting bodies for undergraduate medical education in Nigeria (7).

The study was approved by the Ethics Committee of LAUTECH Teaching Hospital, Oshogbo, Nigeria. The study was conducted in accordance with the STROBE criteria, and the results have been reported accordingly (18,19).

### Questionnaire

We used a self-reported composite questionnaire that was developed from the following sources:

1. The McAllister questionnaire, which contains 11 questions on the sources of information used by the respondents in their practices, 13 questions on their attitude toward EBM, and 8 questions on their confidence in their EBM skills (20).

2. The Zwolsman's barrier questionnaire, from which we included 15 items to explore the self-reported barriers to the use of EBM in practice (21).

3. The Berlin Questionnaire (22) is a common tool for objectively measuring competence in EBM and consists of 15 scenario-based multiple-choice questions, each with only one correct answer. The Berlin Questionnaire tests competence in five of the eight major domains of EBM, and the five items we included covered three domains: magnitude of effect/clinical importance, study design, and internal validity.

Additionally, we included questions on the sociodemographic characteristics, practices, and medical education of the respondents.

Questionnaire validation: Both the original and adapted questionnaires were in English, which is the official language in Nigeria. The adaptation of the composite instrument to our context involved conducting a pilot study with 15 surgeons and resident doctors at the Ekiti State University Teaching Hospital in Ado-Ekiti. Consequently, certain items within the composite questionnaires were reworded based on the feedback from the pilot study. For instance, the statement “Physicians must be able to distinguish methodologically sound from poor research” was revised to read as “Physicians must be able to distinguish research that is methodologically sound from research that is methodologically flawed.” The final instrument comprised 76 questions, some of which underwent rephrasing following the pilot testing.

Scoring of the questionnaires: Both the McAllister and Zwolsman questionnaires use 5-point Likert scale items, scored such that 5 represents “strongly agree” and 1 represents “strongly disagree.” Questions worded negatively were scored in the reverse direction. Correct answers on the Berlin questionnaire were scored as 1, and incorrect answers as 0. Scores were then summed for each participant, with the highest possible total score being 5 and the lowest 0.

Sample size (23): The sample size (n) was calculated according to the formula:

n = [z2 * p * (1 - p) / e2] / [1 + (z2 * p * (1 - p) / (e2 * N))]

Where z = confidence level (α); *P* = proportion of the total with the condition (expressed as a decimal); N = population size; e = margin of error; z = 1.96 for a confidence level of 95%. *P* = 0.7 was based on the 70% proportion of respondents in McAllister’s study who were EBM users (20); N = 311, which is the number of residents and surgeons in the participating hospitals; e = 0.05.

The calculated sample size (with finite population correction) was equal to 159. When a 5% attrition rate was added, the minimum required sample size was 167.

### Statistical analysis

We analyzed the data item by item, or when indicated, by combining all the average ratings of the items in each segment of the questionnaire into an overall mean (summative) score. For example, to get the overall mean McAlister attitude score, the scores of all 13 items in this category were summed together and divided by 13.

For item-by-item analysis, we used the median and interquartile ranges for descriptive statistics, and χ^2^ or Kruskal-Wallis tests for inferential statistics. In some cases, we dichotomized the 5-point scale by including those who “strongly agreed/agreed” into the “agreed” group and those who “strongly disagreed/disagreed/neutral” into the “disagreed” group. Additionally, respondents were categorized based on their reported use of EBM into two groups: self-identified EBM-users (comprising those who “always” or “often” used EBM) and EBM-non-users (including individuals who “sometimes,” “rarely,” or “never” used EBM).

We subjected the summative scores to normality testing with a Kolmogorov-Smirnov test. The *t* test or the Mann-Whitney U test was used to assess the differences in continuous variables between the groups. A χ^2^ test was used for the comparison of categorical variables. The level of statistical significance was 0.05. Data analysis was conducted with SPSS, version 25 (IBM Corp., Armonk, NY, USA). The data set is reposited on the Open Science Framework website (24).

## Results

A total of 185 questionnaires were distributed to surgeons and resident doctors in the five tertiary hospitals. Of these, 16 (8.6%) were excluded due to missing data (when missing data were greater than 60% of the responses) and unengaged responses, leaving 169 (91.4%) questionnaires for analysis. Unengaged responses were those records in which respondents filled in the survey form without paying attention to the questions and answered them consistently with the same number (25). There was no significant difference in the demographics of the included and excluded respondents. The mean age of the respondents was 37.5.2 ± 8.9 years, and the mean postgraduation experience was 10.8 ± 8.4 years.

There were 62 (36.7%) consultant surgeons and 107 (73.3%) resident doctors, including 32 (18.9%) senior registrars and 75 (44.4%) registrars. A total of 32 (18.9%) respondents held academic appointments with universities and were involved in teaching medical students, while the remaining 137 (81.1%) did not have such an appointment. Almost all the specialists were involved in the postgraduate training of resident doctors. There were 120 (71.0%) self-identified EBM users and 49 (29.0%) EBM non-users (Table 1).

**Table 1:** The sociodemographic characteristics and some variables related to evidence-based medicine (EBM) of self-identified EBM users and non-users

Characteristics	EBM user (%) N = 120	EBM non-user (%) N = 49	P
Mean age (years)	37.3	38.0	0.678
Years since graduation (mean)	10.7	10.9	0.861
Sex			
male	111 (94.1)	44 (89.8)	
female	9 (5.9)	5 (10.2)	0.381
Rank			
consultant surgeon	44 (36.7)	18 (36.7)	
trainee	76 (73.3)	31 (73.3)	0.993
Academic appointment at university			
yes	27 (22.5)	4 (8.3)	
no	93 (77.5)	43 (91.7)	0.031
Previous training in EBM			
yes	50 (42.0)	10 (20.4)	
no	69 (58.0)	39 (79.6)	0.008
Spent >1 hr/week reading current literature			
yes	56 (48.3)	14 (29.2)	
no	60 (51.7)	34 (71.8)	0.025

### Knowledge of EBM

To assess the knowledge of EBM, we used the 5-item Berlin Questionnaire. The maximum score was 5 and the minimum was 0. The median score for all respondents was 2 (IQR 1-2). The median score for EBM users was 2 (IQR 1-3) and 1 (IQR 1-2) for EBM non-users (*P* = 0.158).

### Attitudes toward EBM

**Figure 1:**
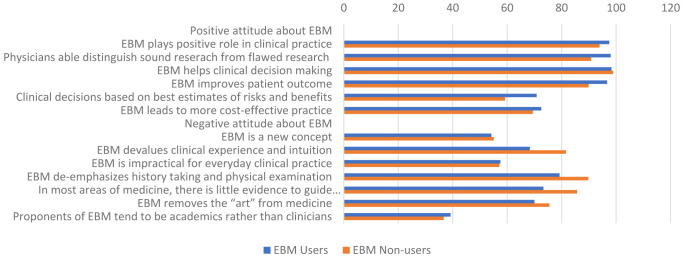
Percentage of respondents in agreement with attitude items of the McAlister Scale. EBM - evidence-based medicine.

The attitude scale of the McAlister questionnaire included a 6-item positive attitude section and a 7-item negative attitude section. Ideally, self-identified EBM users should show a high degree of agreement with positive-attitude statements and low agreement with negative statements. However, the participants’ responses were not consistent (Figure 1). The percentage of respondents agreeing with four of the positive items was higher than 90%. However, the percentage of respondents agreeing with two of the items: “Clinical decisions should be based on the best numerical estimates of risks and benefits” and “EBM leads to more cost-effective practice,” was lower in both groups. Additionally, more than 50% of both EBM users and non-users agreed with the negative-attitude statements, except for the statement “Proponents of EBM tend to be academics rather than front-line clinicians,” which had a lower agreement rate in both groups.

### Confidence in EBM

**Figure 2:**
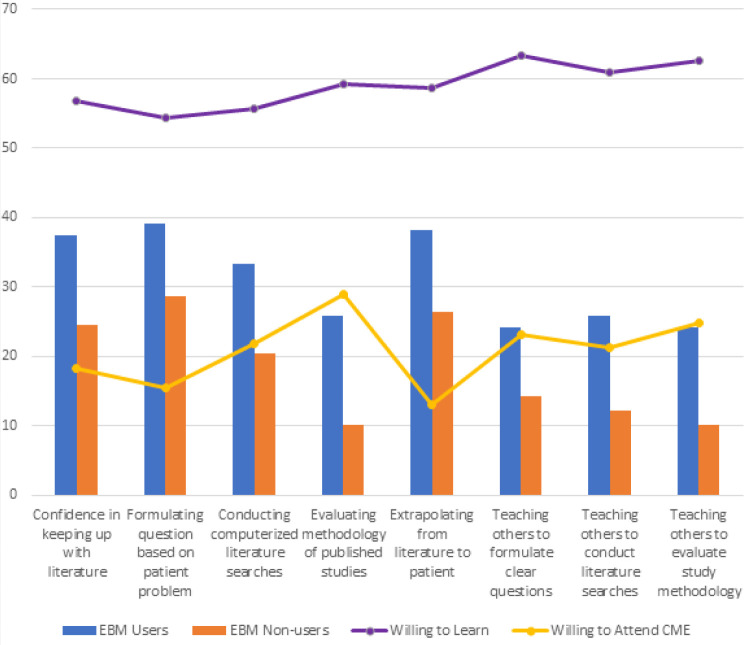
Respondents' confidence in various skillsets of evidence-based medicine (EBM), their willingness to learn and to attend continuing medical education (CME) events on the skillsets in percentage values.

Overall, less than 40% of the respondents felt confident in any of the basic skills of EBM (Figure 2). This was worse for EBM non-users compared with EBM users and was also worse for items related to teaching others. When asked if they would like to learn more about the basic skills of EBM listed in Figure 2, between 54.4% and 63.3% of the participants indicated that they would. However, when asked if they would be willing to attend continuing medical education events devoted to these topics, only 13.0% to 29.0% said they would be willing to do so.

### Sources of information

**Figure 3:**
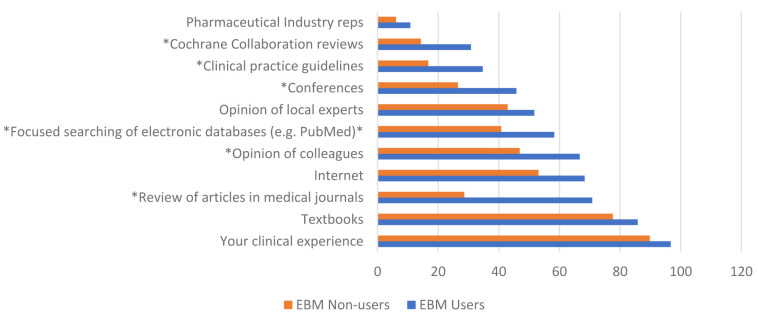
Comparison of evidence-based medicine (EBM) users and non-users in terms of sources of clinical information in percentage values. *P < 0.05.

The sources that practitioners use to look for medical information can indicate EBM usage. Most respondents overwhelmingly relied on traditional sources of information, such as personal experience and textbooks (Figure 3). A minority of respondents frequently used sources recommended by EBM guidelines, such as evidence-based clinical practice guidelines, Cochrane databases, and focused internet searches. However, self-identified EBM users were significantly more likely to use these sources than were non-users. Similarly, those with previous training in EBM and similar techniques were more likely to use EBM-recommended sources than those with no previous training. Less than 10% of respondents reported frequently using information from pharmacy representatives.

Only 18.3% of respondents reported reading professional medical literature for more than four hours per week to stay current with medical literature, while 38.5% only did this occasionally. The median number of medical journals regularly read by respondents was 3 (IQR 2-4), and this was the same for self-identified EBM users and nonusers.

A total of 125 respondents regularly used a personal computer (67, 39.6%), smartphone (18, 14.4%), or tablet (40, 23.7%) at work for email/internet access (85.7%), online searches (52.7%), patient records (5.9%), and research-related activities such as statistical analysis and data entry (37.9%)

### Barriers to EBM

The two most important barriers to EBM identified by the respondents were attitudinal: “I am not interested in searching for the best evidence” (mean 4.17; SD 0.74) and “I am not motivated in working according to the principles of EBM” (mean 3.77; SD 0.93). The next three barriers identified were related to a lack of knowledge and skills, including: “I do not search for clinical evidence because I rely on the formal education I received during specialty training as supplying me with the right knowledge” (mean 3.65; standard deviation 0.96), “I am unsure of what using EBM practically means due to a lack of education in using EBM” (mean 3.54; standard deviation 0.91), and “I do not know when to be satisfied with the answer found when searching for evidence” (mean 3.33; standard deviation 0.949) (Table 2).

**Table 2:** Barriers to the use of evidence-based medicine (EBM)

Barrier to EBM	Mean	Standard deviation
I am not interested in searching for the best evidence	4.17	0.736
I am not motivated in working according to the principles of EBM	3.77	0.932
I do not search for clinical evidence because I rely on the formal education I received during specialty training to supply me with the right knowledge	3.65	0.963
As a result of a lack of education in using EBM, I am unsure of what using EBM practically means	3.54	0.912
When I search for evidence, I do not know when to be pleased with the answer found	3.33	0.949
During consultations, I have sufficient time to work according to the principles of EBM	3.27	0.937
As a result of inexperience with one some of the EBM steps, I do not succeed at using EBM in practice	3.2	1.008
The time I have per patient is insufficient to also search for answers to my questions (according to the principles of EBM)	3.17	1.097
When busy, searching for clinical evidence is not a priority to me	3	1.082
My skills in searching for evidence in databases (ie, PubMed) are sufficient	2.97	1.047
There is enough guidance in my training practice, to support me in using EBM	2.88	0.987
My trainer motivates me to use EBM	2.65	1.022
Formal education stimulates me to use EBM in practice	2.35	0.841
My teacher stimulates me to use EBM	2.33	0.864
When I have a clinical question, I take the initiative to search for an evidence-based answer	2.25	0.799

## Discussion

### Use of EBM

In our study, 97.6% of the respondents had used EBM in their clinical practice in the past, which is consistent with previous studies of pediatricians (90.8%) and obstetricians (96.6%) in Nigeria (10,11). However, only 71% of our study respondents reported always/often employing EBM in their clinical practice (classified as self-identified EBM users). This discrepancy may be because only 35.4% of the respondents in our study had received training in EBM. Previous research has shown that EBM training can increase the adoption of EBM by practitioners in clinical practice (2,3,26). Our study also found that respondents with previous EBM training were significantly more likely to use EBM in their practice than those without such training.

### Attitude toward EBM

The respondents’ attitudes toward EBM were ambivalent. On the one hand, most of the respondents agreed with positively worded statements about EBM, but more than 50% also agreed with negatively worded statements. This contrasts with the findings of a study by McAlister et al among Canadian general practitioners, in which most respondents agreed with positive statements about EBM and less than 30% agreed with negative statements (20). However, our findings are consistent with those of Kitto et al, who found that surgeons in Australia “demonstrate ambivalent and contradictory attitudes towards EBM in surgical practice” (9). This ambivalence toward EBM may be a reflection of surgeons' reluctance to embrace EBM in the same way as their counterparts in internal medicine. Surgeons often believe that their work involves more than just science and that there is an art to surgery that cannot be captured in EBM guidelines (9). More than many other medical specialties, surgery may also be more dependent on contingencies. Pope has identified three categories of contingency (case, surgeon, and external), which may help explain some of the difficulties that surgeons face when adopting EBM in their clinical practice (27). This contingent aspect of surgery, which is a unique configuration of individual case, surgeon and external factors, requires sometimes unique practical and technical solutions that may not be covered by practice guidelines (28). Griffith et al also found that the choice of surgical procedure for a patient depends on the patient’s diagnosis, the expertise of the surgeon, and the hospital (29). As a fallout of this conflict, many authors have advocated for a synergy between the art and science as a basis for providing evidence-based guidelines for surgeons (30). Monash et al have also advocated for the use of mixed-method research, which combines quantitative and qualitative methods to consider the cultural, clinical, and behavioral aspects of surgical practice (9).

### Sources of information and confidence in EBM

It has been said that “if you read two medical papers every day, then in a year, you are two centuries behind in your reading.” (27) At the current rate, medicine advances in knowledge at the speed of roughly doubling data in 48 months. Trying to keep up with this is not only impossible, but for most practitioners, it is also unnecessary. Primarily, practitioners need to stay updated within their own specialties. Additionally, not all published material in a given specialty requires review. Whenever new research in a specialty is published, it goes through a chain of reviews. Some compare the data and others validate their practical applicability. Some compare or combine the study with others. Once all that is done, there is a guideline. And that is what physicians need to read. Thus, for physicians to keep abreast of current developments in their field, they need to have access to, and be able to understand sources of current medical information such as journals, practice guidelines, and Cochrane databases.

However, in this study, less than 20% of respondents reported reading professional medical literature for more than four hours per week. The most common sources of professional information cited by the surgeons in our study were their clinical experiences and textbooks. Textbooks can be useful sources of information on history, examination, and some investigations, but they may be outdated when it comes to treatment choices, as they are often 5-10 years old by the time they are published (20,31). Clinical experience may be even less reliable as a source of current information.

Furthermore, fewer than 40% of the respondents in our study felt confident using EBM. This low level of confidence was consistent across different skills related to EBM. Despite this lack of confidence, most respondents were open to learning more about EBM. However, there was a notable discrepancy in their willingness to invest in such training, with only a minority willing to attend paid continuing medical education events. This gap suggests barriers such as time, cost, or perceived relevance might deter participation in formal training. It also indicates a potential preference for more flexible, informal learning methods.

One approach to improving the use of evidence-based sources of information by surgeons is through EBM training. Consistent with the results reported by Balajić et al, respondents in our study who had prior training in EBM and related techniques were more likely to use evidence-based sources of medical information in their clinical practice (2). Therefore, offering EBM training opportunities for Nigerian surgeons is likely to increase their adoption of evidence-based practice. However, considering our participants' reluctance to pay for EBM training, this training should ideally begin at the undergraduate level, incorporated into the minimum standards for basic medical education as set by the National University Commission and the Medical and Dental Council of Nigeria (32). Additionally, the postgraduate medical colleges in Nigeria should also include EBM in their curricula. Lastly, stakeholders in the Ministry of Health should consider implementing a national EBM guideline program similar to the UK's NICE program.

### Barriers to EBM

In contrast to the findings of McAlister et al, who identified lack of knowledge and skills as the main barriers to the adoption of EBM among a group of Canadian general practitioners, the two most important barriers identified by surgeons in our study were attitudinal. This is not surprising, as previous research has shown that surgeons are more resistant to adopting EBM in their practice than other specialists (2,16,18). The next three most important barriers identified in our study were related to knowledge and skills. This is also reflected in the poor performance of the respondents on the Berlin knowledge questionnaire, whose main strength is that it evaluates applied knowledge. The results showed that most of the surgeons in our study lacked practical knowledge in many domains of EBM. However, the good news is that most of the respondents would be willing to learn EBM, and as previously mentioned, policymakers and stakeholders should provide the necessary framework to make this possible.

### Limitations of the study

This survey's limitation is that it evaluates self-reported rather than actual EBM practices among Nigerian surgeons. This may result in an overestimation of the prevalence of EBM practices, as respondents may report what they believe to be the correct answer rather than their actual practices. Consequently, the level of EBM reported in this survey may be higher than the actual level of practice.

### Conclusion

In conclusion, while surgeons in Nigeria have a reasonable level of awareness and use of EBM, their knowledge and confidence in its application is low. This is likely due to the absence of a formal platform in the country for surgeons to learn about and improve their knowledge of EBM. In other words, there is no active encouragement of the use of EBM among surgeons in Nigeria. The main barriers to EBM usage appear to be both attitudinal and knowledge/skill-related, and these barriers can be addressed by setting up a national framework for the implementation of EBM. This could include incorporating EBM into undergraduate and postgraduate curricula and developing national guidelines for EBM similar to the UK's NICE program.
